# Association of Obesity, Diabetes, and Alcohol Use With Liver Fibrosis Among US Adults With Hepatitis C Virus Infection

**DOI:** 10.1001/jamanetworkopen.2021.42282

**Published:** 2022-03-18

**Authors:** Alexandra L. Migdal, Ram Jagannathan, Emad Qayed, Kenneth Cusi, Rozalina G. McCoy, Francisco J. Pasquel, Lesley S. Miller

**Affiliations:** 1 Division of Endocrinology, Department of Medicine, Emory University School of Medicine, Atlanta, Georgia; 2 Division of Hospital Medicine, Department of Medicine, Emory University School of Medicine, Atlanta, Georgia; 3 Division of Gastroenterology, Department of Medicine, Emory University School of Medicine, Atlanta, Georgia; 4 Division of Endocrinology, Diabetes, and Metabolism, Malcom Randall Veterans Affairs Medical Center, Gainesville, Florida; 5 Division of Community Internal Medicine, Department of Medicine, Mayo Clinic, Rochester, Minnesota; 6 Mayo Clinic Robert D. and Patricia E. Kern Center for the Science of Health Care Delivery, Rochester, Minnesota; 7 Division of General Internal Medicine, Department of Medicine, Emory University School of Medicine, Atlanta, Georgia

## Abstract

This cross-sectional study examines the association of obesity, diabetes, and alcohol use with liver fibrosis among treatment-naive US adults with hepatitis C virus infection seen at a safety-net hospital in Atlanta, Georgia.

## Introduction

Chronic hepatitis C virus (HCV) infection can lead to liver inflammation, fibrosis, and ultimately cirrhosis and hepatocellular carcinoma (HCC). The results of previous studies suggest that older age, HIV infection, obesity, and diabetes are associated with advanced stages of fibrosis in HCV.^1,2,3,4,5^ However, these risk factors are likely interdependent and potentially exacerbated by other conditions that may contribute to liver fibrosis.^6^ Identifying patients with HCV who are at risk for advanced fibrosis is important for evidence-based, efficient HCC screening practices. We therefore examined the association of HCV, obesity, diabetes, and alcohol use with liver fibrosis by using electronic health records (EHRs) from a large database of patients with HCV seen at a safety-net hospital in Atlanta, Georgia.

## Methods

This cross-sectional study was approved by the Emory University Institutional Review Board. Informed consent was waived because the research involved no more than minimal risk, the research could not practicably be carried out without the requested waiver or alteration, and the waiver would not adversely affect the rights and welfare of the subjects. The study followed the Strengthening the Reporting of Observational Studies in Epidemiology (STROBE) reporting guideline.

We conducted a retrospective, cross-sectional analysis of treatment-naive adults with HCV seen at Grady Memorial Hospital Liver Clinic in Atlanta, Georgia, who had index ultrasound elastography studies performed between January 1, 2018, and December 31, 2019. All data within approximately 12 months of ultrasound elastography were ascertained from EHRs. Continuous data are presented as the mean (SD) or median (IQR), and categorical data are presented as frequency distributions. To compare continuous variables, we conducted *F* tests or nonparametric tests for variables with nonnormal distributions and χ^2^ tests for categorical variables. Data were stratified by diabetes and obesity (body mass index > 30, calculated as weight in kilograms divided by height in meters squared). The outcome variables of interest were fibrosis severity (F0/F1 to F4 stage) and steatosis severity (S0 to S3 stage). Because racial differences have been noted in liver-related outcomes such as steatosis and fibrosis, we gathered self-reported race and ethnicity data from the EHR. To explore effect modification, we investigated whether the association of diabetes with fibrosis and steatosis differed according to alcohol use, adding a multiplicative interaction term between diabetes status and alcohol use. Additional details are provided in the eMethods in the Supplement.

## Results

We identified 965 patients who underwent ultrasound elastography. Five patients were excluded because of missing data, resulting in a final sample of 960 patients with a mean (SD) age of 58.3 (10.2) years. Of these 960 patients, 632 (65.8%) were men, 761 (79.3%) were Black, 247 (25.7%) had obesity, 231 (24.0%) had diabetes, and 260 (27.1%) had a history of alcohol use. Overall fibrosis scores were as follows: 501 patients (52.2%) had F0 to F1 (no scarring to mild scarring), 212 patients (22.1%) had F2 (moderate scarring), 80 patients (8.3%) had F3 (severe scarring), and 167 patients (17.4%) had F4 (advanced scarring [cirrhosis]). Steatosis scores were as follows: 622 patients (64.8%) had S0 (none), 117 patients (12.2%) had S1 (mild), 111 patients (11.6%) had S2 (moderate), and 110 patients (11.5%) had S3 (severe). Compared with patients without obesity or diabetes, patients with obesity or a combination of obesity and diabetes had higher rates of S3 steatosis (5.7% vs 22.4% vs 34.1%; *P* < .001; Table).

**Table.** Clinical Characteristics of Patients With Hepatitis C Stratified by Obesity and Diabetes

Variable	No. of patients (%)					*P* value
	Overall (n = 960)	No obesity or diabetes (n = 564)	Obesity but no diabetes (n = 165)	Diabetes but no obesity (n = 149)	Obesity and diabetes (n = 82)	
Demographic						
Mean age, y^a^	58.3 (10.2)	57.7 (10.8)	55.4 (10.3)	62.3 (6.9)	60.8 (6.6)	<.001
Men	632 (65.8)	409 (72.5)	82 (49.7)	101 (67.8)	40 (48.8)	<.001
Race and ethnicity						<.001
Black	761 (79.3)	421 (74.6)	129 (78.2)	134 (89.9)	77 (93.9)	
Hispanic	13 (1.4)	4 (0.7)	3 (1.8)	5 (3.4)	1 (1.2)	
White	167 (17.4)	128 (22.7)	29 (17.6)	7 (4.7)	3 (3.7)	
Other	19 (2.0)	11 (2.0)	4 (2.4)	3 (2.0)	1 (1.2)	
Mean BMI^a^	26.8 (5.8)	24.1 (3.4)	34.7 (4.3)	24.6 (3.7)	34.1 (3.5)	<.001
Lifestyle habit						
Smoking	410 (42.7)	234 (41.5)	64 (38.8)	77 (51.7)	35 (42.7)	.99
Alcohol	260 (27.1)	172 (30.5)	36 (21.8)	34 (22.8)	18 (22.0)	.044
Comorbidity						
Hypertension	670 (69.9)	343 (60.8)	116 (70.3)	131 (87.9)	80 (97.6)	<.001
Dyslipidemia	303 (31.6)	124 (22.0)	34 (20.6)	94 (63.1)	51 (62.2)	<.001
CHF	119 (12.4)	58 (10.3)	20 (12.1)	26 (17.4)	15 (18.3)	.04
CAD	89 (9.3)	51 (9.0)	13 (7.9)	16 (10.7)	9 (11.0)	.782
COPD	171 (17.8)	94 (16.7)	30 (18.2)	29 (19.5)	18 (22.0)	.62
CKD	154 (16.0)	73 (12.9)	20 (12.1)	44 (29.5)	17 (20.7)	<.001
MI	45 (4.7)	26 (4.6)	8 (4.8)	7 (4.7)	4 (4.9)	.999
PAD	106 (11.0)	52 (9.2)	19 (11.5)	29 (19.5)	6 (7.3)	.003
Outcome						
Mean fibrosis score^a^	9.8 (9.3)	9.4 (9.5)	9.8 (9.5)	10.8 (8.5)	10.4 (8.3)	.39
Fibrosis stage^b^						<.001
F0 to F1	501 (52.2)	336 (59.6)	79 (47.9)	57 (38.3)	29 (35.4)	
F2	212 (22.1)	113 (20.0)	39 (23.6)	35 (23.5)	25 (30.5)	
F3	80 (8.3)	31 (5.5)	19 (11.5)	18 (12.1)	12 (14.6)	
F4	167 (17.4)	84 (14.9)	28 (17.0)	39 (26.2)	16 (19.5)	
Steatosis stage^c^						<.001
S0	622 (64.8)	426 (75.5)	67 (40.6)	98 (65.8)	31 (37.8)	
S1	117 (12.2)	62 (11.0)	21(12.7)	25 (16.8)	9 (11.0)	
S2	111 (11.6)	44 (7.8)	40 (24.2)	13 (8.7)	14 (17.1)	
S3	110 (11.5)	32 (5.7)	37 (22.4)	13 (8.7)	28 (34.1)	

Abbreviations: BMI, body mass index (calculated as weight in kilograms divided by height in meters squared); CAD, coronary artery disease; CHF, congestive heart failure; CKD, chronic kidney disease; COPD, chronic obstructive pulmonary disease; MI, myocardial infraction; PAD, peripheral artery disease.

^a^ Values are presented as the mean (SD).

^b^ Fibrosis stages are as follows: F0 to F1, no scarring to mild scarring; F2, moderate scarring; F3, severe scarring; and F4, advanced scarring (cirrhosis).

^c^ Steatosis stages are as follows: S0, none; S1, mild; S2, moderate; and S3, severe.

Diabetes was independently associated with advanced fibrosis (odds ratio [OR], 1.76 [95% CI, 1.29-2.39]) and steatosis (OR, 1.44 [95% CI, 1.04-2.02]) in the fully adjusted model (Figure). Alcohol use was independently associated with fibrosis (OR, 1.43 [95% CI, 1.08-1.89]) and steatosis (OR, 1.40 [95% CI, 1.03- 1.90]), as was obesity status (fibrosis OR, 1.36 [95% CI, 1.02-1.80]; steatosis OR, 4.78 [95% CI, 3.52-6.50]). Similar findings were observed in a stratified analysis by sex. There were no significant interactions between diabetes status and alcohol use with fibrosis (*P* = .24) and steatosis (*P* = .99) severity outcomes.

**Figure.**
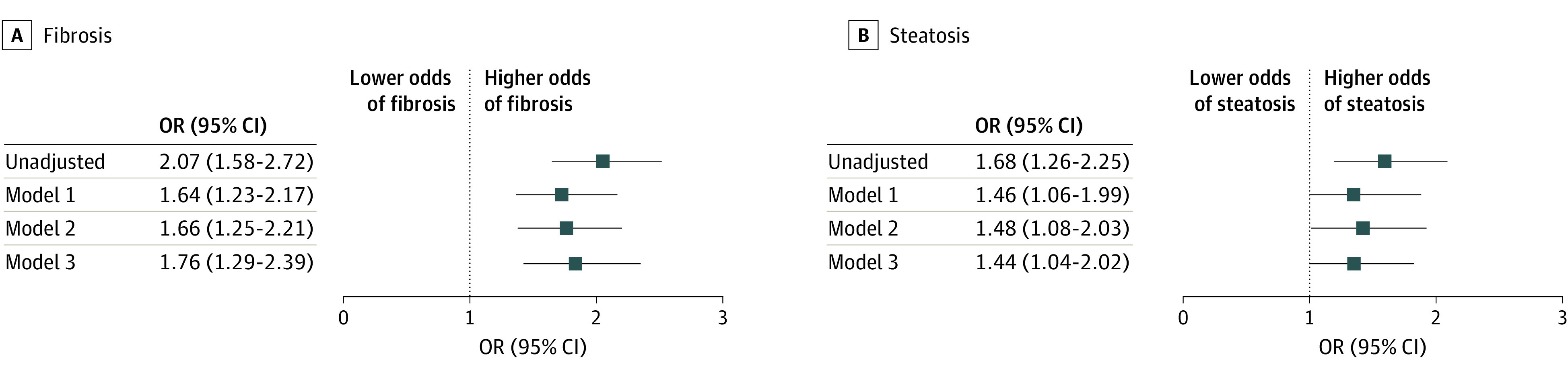
Association of Diabetes With Advanced Fibrosis and Steatosis Among 960 Patients With Hepatitis C Virus Infection Model 1 includes age (continuous), sex (male or female), obesity (yes or no), and race (Black, Hispanic, White, and other). Model 2 includes the variables in model 1 plus alcohol use (yes or no). Model 3 includes the variables in model 2 plus hypertension and dyslipidemia (both yes or no). OR denotes odds ratio.

## Discussion

Liver fibrosis is an important precursor of chronic liver disease and its complications. Findings from this large real-world evaluation of treatment-naive patients with HCV, focusing specifically on patients with a high prevalence of obesity, diabetes, and alcohol use in underserved populations, revealed an independent relationship between diabetes and liver fibrosis severity. Alcohol use was also associated with worse fibrosis, but no interaction was noted between diabetes and alcohol use. One limitation of this study is that a directional relationship could not be determined because of the cross-sectional nature of the analysis.

Our findings suggest an urgent need to investigate the interaction of multiple risk factors and the progression of liver disease to help inform evidence-based liver cancer screening strategies for individuals at highest risk.

## Notes

Supplement. **eMethods. **Ordinal Logistic Regression Analysis Modeling of the Association of Diabetes With Fibrosis or Steatosis Outcome Severity
eReferences

## References

[1] Afsari A, Lee E, Shokrani B, . Clinical and pathological risk factors associated with liver fibrosis and steatosis in African-Americans with chronic hepatitis C. Dig Dis Sci. 2017;62(8):2159-2165. doi:10.1007/s10620-017-4626-7 28612194 PMC5706543

[2] Poynard T, Ratziu V, Charlotte F, Goodman Z, McHutchison J, Albrecht J. Rates and risk factors of liver fibrosis progression in patients with chronic hepatitis c. J Hepatol. 2001;34(5):730-739. doi:10.1016/S0168-8278(00)00097-0 11434620

[3] Ong JP, Younossi ZM, Speer C, Olano A, Gramlich T, Boparai N. Chronic hepatitis C and superimposed nonalcoholic fatty liver disease. Liver. 2001;21(4):266-271. doi:10.1034/j.1600-0676.2001.021004266.x 11454190

[4] Papatheodoridis GV, Chrysanthos N, Savvas S, . Diabetes mellitus in chronic hepatitis B and C: prevalence and potential association with the extent of liver fibrosis. J Viral Hepat. 2006;13(5):303-310. doi:10.1111/j.1365-2893.2005.00677.x 16637860

[5] Monto A, Alonzo J, Watson JJ, Grunfeld C, Wright TL. Steatosis in chronic hepatitis C: relative contributions of obesity, diabetes mellitus, and alcohol. Hepatology. 2002;36(3):729-736. doi:10.1053/jhep.2002.35064 12198667

[6] Blomdahl J, Nasr P, Ekstedt M, Kechagias S. Moderate alcohol consumption is associated with advanced fibrosis in non-alcoholic fatty liver disease and shows a synergistic effect with type 2 diabetes mellitus. Metabolism. 2021;115:154439. doi:10.1016/j.metabol.2020.154439 33246008

