# Supplementary material for: Association of Obesity, Diabetes, and Alcohol Use With Liver Fibrosis Among US Adults With Hepatitis C Virus Infection
Source: JAMA Netw Open. 2022 Mar 18;5(3):e2142282. doi: 10.1001/jamanetworkopen.2021.42282 (PMC8933742; doi:10.1001/jamanetworkopen.2021.42282)
Supplement: Supplement. — eMethods. Ordinal Logistic Regression Analysis Modeling of the Association of Diabetes With Fibrosis or Steatosis Outcome Severity eReferences [file jamanetwopen-e2142282-s001.pdf]

Supplementary Online Content

Migdal AL, Jagannathan R, Qayed E, et al. Association of obesity, diabetes, and alcohol use with liver fibrosis among US adults with hepatitis C virus infection. *JAMA Netw Open*. 2022;5(3):e2142282. doi:10.1001/jamanetworkopen.2021.42282

eMethods. Ordinal Logistic Regression Analysis Modeling of the Association of Diabetes With Fibrosis or Steatosis Outcome Severity

eReferences

This supplementary material has been provided by the authors to give readers additional information about their work.

© 2022 Migdal AL et al. *JAMA Network Open*.

eMethods. Ordinal Logistic Regression Analysis Modeling of the Association of Diabetes With Fibrosis or Steatosis Outcome Severity

All data analysis was performed in R (R Core Team, 2020, version: 4.0.2). The ordered logistic regression models were fitted using the "polr" function in the "MASS" package [1] to ascertain the association between diabetes status (yes/no) and the ordered categories of fibrosis and steatosis outcomes. The unadjusted and multivariable ordered logistic regressions were used to evaluate the association between diabetes status (yes/no) and fibrosis or steatosis severity. For each fibrosis or steatosis severity group, we assigned a number in an ascending order to represent the increasing severity in the ordered logistic model. We initially adjusted the models for age, gender, obesity, and race (model 1) and in addition for alcohol use (model 2), and with hypertension and dyslipidemia (model 3).

Model selection

Model-1 was adjusted for age (per SD), sex (male/female), obesity (body mass index (BMI)> 30kg/m^2 (yes) vs. BMI< 30kg/m^2 (no), and race (White, Black, and others);

Model 2 was adjusted for the variables in Model-1 in addition to alcohol use (yes/no);

Model-3 was adjusted with hypertension (yes/no) and dyslipidemia status (yes/no) in addition to the variables in Model-2.

Covariables included in the models were either continuous (per SD changes for age) or categorical. The primary analysis included the overall sample (n=960); secondary analyses were conducted in sex-specific subgroups.

Interaction assessment

To assess the interaction between the diabetes status (yes/no) and alcohol use (yes/no) on the subsequent association with fibrosis and steatosis severity was explored by including a multiplicative interaction term in the ordered logistic regression analysis models. We did not observe any evidence of multicollinearity between covariates for any of the fitted models (the variance inflation factor (VIF)<2 for all the independent variables [2]. We tested for parallel regression assumption for ordinal models with the "brant" function in the "brant" package [3]. This assumption confirms that the association between each pair of outcome categories is the equivalent and should be fulfilled to implement ordinal models. The Brant test showed that the

© 2022 Migdal AL et al. *JAMA Network Open*.

parallel regression assumption holds, which should be fulfilled to employ ordered logistic models for our analyses.

eReferences

1. Ripley B, Venables B, Bates DM, Hornik K, Gebhardt A, Firth D and Ripley MBJCr. Package ‘mass’. 2013.

2. Fox J and Weisberg S. An R companion to applied regression: Sage publications; 2018.

3. Schlegel B, Steenbergen M, Schlegel MB and Imports M. Package brant. 2017.

© 2022 Migdal AL et al. *JAMA Network Open*.
